# Munificent Gifts to Medical Institutions by M. Orfila

**Published:** 1853-04

**Authors:** 


*Munificent Gifts to Medical Institutions by M. Orfila.*— M. Orfila recently read the following letter before the Academy of Paris:

I do not wait, as is generally the rule, till death has removed me from among you, to assign the sum of $24,000 to different public establishments. I have two reasons for acting thus: first, because it is of some importance that the institutions to which I refer should as soon as possible reap the benefit of the donations which I am offering; and secondly, because I thought that my presence would be of some use to overcome any difficulties which might arise during the carrying out of my plan; or perhaps in order to modify the latter, if the necessity of doing so were clearly demonstrated. I shall not attempt to enter into the reasons which have induced me to give the preference to certain institutions over others; it will be sufficient for me to state that, by giving $12,000 to Government for the complet'on of the museum which bears my name, it is my intention to endow France with a scientific collection which will be unparalleled, and also to afford students in medicine a new proof of the sympathy and good will with which I have always regarded them. I am also anxious to show them how grateful I feel for the very flattering attention they invariably have given to my lectures for the last thirty-four years. I am anxious, for this reason, that no misapprehension should exist regarding my motives, and have directed the following inscription to be placed over the principal entrance to the museum:

To Students in Medicine.—I founded this Museum, in 1845, for promoting medical studies, and solely to be useful to yourselves.—Orfila.

I have thought it right to found a small annui'y in favor af the keeper, who has always rigidly attended to his duties. I also institute two prizes, the one to be given by the Academy of Medicine ($400), the other by the School of Pharmacy ($200), on subjects which have fixed my attention all through life. I have thus no other ambition but that of serving a science to which I have always remained faithful, without allowing myself to be led astray by politics. I give to the Preparatory Schools of Bordeaux and Angers, $200 to the former, and $440 to the latter, to show how I approve of this kind of schools, which were organized upon a proposal of mine. To the Benevolent Medical Association of the Department of

Seine I give $80 a year, in proof of the high estimation in which I hold this society, which I am proud of having founded in 1833.

M. Orfila mentioned various other acts of kindness and benevolence of smaller importance, and received, at the end of his discourse, the hearty and unanimous applause of the members present. The Academy have decided that thanks should be tendered to M. Orfila by a deputation; and the medical press are calling a meeting for the same purpose.—*London Lancet.*

